# A Distance-Based Energy Aware Routing Algorithm for Wireless Sensor Networks

**DOI:** 10.3390/s101009493

**Published:** 2010-10-21

**Authors:** Jin Wang, Jeong-Uk Kim, Lei Shu, Yu Niu, Sungyoung Lee

**Affiliations:** 1 Department of Computer Engineering, Kyung Hee University, Suwon, Korea; E-Mails: wangjin@oslab.khu.ac.kr (J.W.); niuyu@oslab.khu.ac.kr (Y.N.); 2 Department of Energy Grid, Sang Myung University, Seoul, Korea; E-Mail:jukim@smu.ac.kr (J.-U.K.); 3 Department of Multimedia Engineering, Osaka University, Japan; E-Mail: lei.shu@ist.osaka-u.ac.jp (L.S.)

**Keywords:** wireless sensor networks, routing, hotspot, hop number, energy efficiency, network lifetime

## Abstract

Energy efficiency and balancing is one of the primary challenges for wireless sensor networks (WSNs) since the tiny sensor nodes cannot be easily recharged once they are deployed. Up to now, many energy efficient routing algorithms or protocols have been proposed with techniques like clustering, data aggregation and location tracking *etc.* However, many of them aim to minimize parameters like total energy consumption, latency *etc.*, which cause hotspot nodes and partitioned network due to the overuse of certain nodes. In this paper, a Distance-based Energy Aware Routing (DEAR) algorithm is proposed to ensure energy efficiency and energy balancing based on theoretical analysis of different energy and traffic models. During the routing process, we consider individual distance as the primary parameter in order to adjust and equalize the energy consumption among involved sensors. The residual energy is also considered as a secondary factor. In this way, all the intermediate nodes will consume their energy at similar rate, which maximizes network lifetime. Simulation results show that the DEAR algorithm can reduce and balance the energy consumption for all sensor nodes so network lifetime is greatly prolonged compared to other routing algorithms.

## 1. Introduction

Wireless sensor networks (WSNs) have received lots of attention in recently years due to their wide applications like military and disaster surveillance, industrial product line monitoring, agricultural and wildlife observation, healthcare, smart homes, *etc.* [1]. Cheap and tiny sensor nodes are usually randomly deployed in a physical environment to be monitored and they will transmit their collected data to certain remote sink node (or base station) in an autonomous and unattended manner.

Energy efficiency and balancing is one of the primary challenges to the successful application of WSNs since the sensor nodes are powered with limited batteries and they cannot be easily recharged once deployed. Up to now, many energy efficient routing algorithms or protocols have been proposed with techniques like clustering, data aggregation, multi-path and location tracking, *etc.*, as can be seen from related work. However, many of them aim to minimize parameters like total energy consumption or delay during the routing process, which might cause some hotspot nodes as well as a partitioned network due to the overuse of certain nodes. Since the network lifetime is usually defined as the time when the first node dies from lack of energy, huge amounts of energy will be wasted by the remaining sensor nodes.

Hop number and hop distance have a very important impact on many network metrics like energy consumption, routing overhead, interference, latency, *etc.* Intuitively, if the hop number is too large, the energy consumption can be reduced at the cost of long end-to-end latency and large control overhead. If the hop number is too small (e.g., direct transmission), the latency will be very small while the energy consumption can be very large due to the long distance wireless communication nature. Therefore, an optimal hop number with suitable individual distance(s) needs to be deduced as a tradeoff in order to achieve energy reduction and energy balancing.

Hotspot problems are caused by an unbalanced energy consumption among the sensors [2–5]. It is a big challenge to overcome under the random and dynamic topology of WSNs. Besides, the routing scheme and traffic pattern vary under different applications, which add to the difficulty of energy balancing and will usually lead to hotspot nodes and partitioned networks. For example, when all sensors use direct transmission, the nodes far away from sink node will die earlier since the energy consumption is proportional to the fourth order of the long distance. Meanwhile nodes close to the sink node will have much residual energy. On the other hand, when multi-hop transmission is used, nodes near a sink node will have more traffic to forward and will die quickly while nodes far from a sink node will have much remaining energy by using short distance multi-hop transmission. This hotspot issue also appears in many other energy efficient routing algorithms or protocols like those described in [6–8], *etc.*

To effectively alleviate the hotspot problem, we need to balance energy consumption among all sensors by considering factors like manner of transmission, traffic patterns, hop number and distance, *etc.* Based on our previous work in [5], we find that the final residual energy is still not well balanced when the first node dies, even though the energy consumption is largely reduced and the network lifetime is extended. The main reason is that in [5] we tried to minimize the total energy consumption on each route so that some nodes close to sink node are overused, which causes a hotspot problem.

In order to alleviate the hotspot problem, we study the energy consumption under different energy and traffic models and aim to let all sensor nodes consume their energy at similar rate. In other words, we are not trying to $\mathrm{Min}(\sum_{i=1}^{n}E_{i})$ for the *n*-hop route but rather to equalize *E_1_ ≈ E_2_ ≈* ⋯ *≈ E_n_* for the involved intermediate *n* nodes with proper individual distance *d_i_*. Here, *E_i_* is the energy consumption for each individual node.

The main objective in this paper is to prolong network lifetime via an energy efficient and balancing routing algorithm and our contributions are listed as below:

1. Given the source to sink node distance *d*, the optimal multi-hop number and the corresponding individual distance *d_i_* can be determined based on the theoretical analysis of energy consumption under event based and time based traffic model.
2. Based on (1), a Distance-based Energy Aware Routing (DEAR) algorithm is proposed which consists of route setup and route maintenance phases. The distance factor is treated as the first parameter during the routing process and the residual energy factor is the second parameter to be considered. The DEAR algorithm can balance energy consumption for all sensor nodes and consequently prolong the network lifetime.
3. Simulation results and comparisons are provided with discussion details.

The rest of the paper is organized as follows. Section 2 introduces some related work of energy efficient routing algorithms. In Section 3 we present relevant network, traffic and energy models. In Section 4, the details of our DEAR algorithm are described based the theoretical deduction and numerical analysis under different models. Performance evaluation and comparison are given in Section 5 and Section 6 concludes this paper.

## 2. Related Work

Up to now, many techniques have been proposed to improve the energy efficiency in different layers of WSNs. For example, the technique of coding in the PHY layer, the scheduling mechanism of “active/idle/sleeping” in the MAC layer as well as cross-layer optimization methods can reduce energy consumption to a certain degree. In this paper, we focus on energy efficient routing in the network layer.

**Figure 1.**
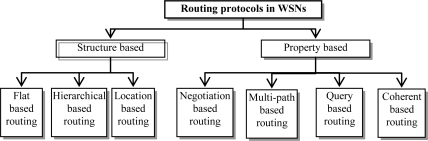
Routing protocols in sensor networks: A taxonomy.

Many energy efficient routing protocols or algorithms have been proposed for WSNs recently. In this section, we first introduce some traditional energy efficient routing algorithms which are listed in the left side of Figure 1 (similar to [9]). Then another kind of energy efficient routing algorithms based on soft computing techniques such as genetic algorithm (GA), ant colony optimization (ACO) and swarm intelligence (SI) are introduced. Finally a few recent studies from hop-based or distance-based energy aware routing algorithms are provided.

### 2.1. Traditional Energy Efficient Routing

As is shown in the left side of Figure 1, the traditional routing protocols in WSNs can be classified into flat (or data centric [10]), hierarchical and location based routing. Among flat routing protocols, SPIN (Sensor Protocols for Information via Negotiation [11]) can be viewed as the first data-centric routing protocol which utilizes the data negotiation method among sensor nodes to reduce data redundancy and save energy. Direct Diffusion [12] is another representative data-centric routing protocol for WSNs. The data generated by sensor nodes is named by attribute-value pairs. Once a sink node inquires certain type of information (like four-legged animals in a certain area), it will send a query and the observed data can get aggregated and then be transmitted back to the sink node. In addition, the load balancing can be achieved by forwarding the data on different paths based on probability. Rather than always using the lowest energy paths, the authors in [13] use sub-optimal paths occasionally so that the network lifetime is increased by 40% compared to [12].

Hierarchical routing protocols [6–8,14] are very suitable for WSNs since they can not only provide good scalability for hundreds or thousands of sensors, but also perform data aggregation by cluster head within each cluster. LEACH [6,14] is one of the most famous hierarchical routing protocols for WSNs. It can prolong network lifetime up to 8-fold more than other ordinary routing protocols like direction transmission and minimum transmission energy routing protocols. However, the 5% of cluster head nodes are randomly chosen and the cluster head nodes use direct transmission to the sink node therein. PEGASIS [7] is viewed as an improved version of LEACH. It is a chain based routing protocol which can save more energy compared to LEACH. The message can get aggregated along the chain and finally be sent to sink node via direct transmission by one random node on the chain. The main shortcoming is that PEGASIS requires global knowledge of the whole network. HEED clustering protocol [8] considers the residual energy as the primary parameter and a secondary parameter like node’s degree during the selection of cluster head. It can not only minimize the control overhead but also prolong network lifetime than other clustering algorithms like LEACH since the cluster heads are well distributed. Besides, it does not need global knowledge of the whole network and all intelligent decisions are made locally by sensor nodes.

Location-based routing protocols [15,16] can get location information through global positioning system (GPS) devices or certain estimation algorithms based on received signal strength (RSS). Once the location information is known, the energy consumption can be largely reduced through power control techniques and communication overhead can also be reduced. MECN [15] provides a minimum energy network for WSNs under the support of low power GPS. The authors in [16] make an extension of [15] by considering possible obstacles between any pair of communication nodes.

### 2.2. Soft Computing Based Energy Efficient Routing

Due to the dynamic nature of WSNs, the optimization of network metrics like shortest path, minimal energy consumption can be viewed as a combinatorial optimization problem which is hard to solve. Since soft computing based techniques can dynamically adjust their parameters during the search of the optimal value, it is very suitable to be used to solve these kinds of dynamic optimization problems, such as NP-hard problems.

A multi-path routing protocol based on dynamic clustering and ant colony optimization (ACO) is proposed in [17] so as to reduce energy consumption and maximize network lifetime. The ACO technique is used during the search for multi-paths between cluster head and sink node. In [18], an improved version of LEACH [6] is presented to improve energy efficiency and system stability where genetic algorithm (GA) is utilized during the selection of cluster head nodes. GA can also be applied to minimize data latency once the number of gateways and their positions are determined [19]. In [20], each swarm agent can carry and exchange the residual energy information during the route selection process in order to maximize the network lifetime in ad hoc and sensor networks.

### 2.3. Hop-Based Energy Efficient Routing

In hop-based routing, the metric of (optimal) hop number or corresponding individual distance is treated as the primary factor in order to achieve energy efficiency during routing process. It can be seen that the factor of hop number or hop distance is not specifically proposed and addressed by most of the energy efficient routing protocols above, when in fact, it has very important impact on many network metrics like energy consumption, latency, routing overhead, interference, *etc.* [21].

The authors in [22] present some pioneering work by studying different energy models under general wireless network environment. The authors in [6,14] consider different energy consumption for source and intermediate node and each cluster head uses direct transmission under their small scale network environment. The authors in [2] study selection of transmission manner from probability point of view. They present a probability of *P_i_* to transmit data through multi-hop manner and a probability (1 − *P_i_*) to transmit through single hop manner to sink node. The authors in [3] also study the energy consumption under both single hop and multi-hop transmission manners. They claim that the preference of multi-hop routing to single hop routing depends on source to sink distance and reception cost. The author in [4] proposes a Multihop/Direct Forwarding scheme to split data traffic into two branches by using either direct or multihop transmission to achieve good energy efficiency and network lifetime performance. In our previous work [5], we propose a Hop-based Energy Aware Routing (HEAR) algorithm which can largely reduce energy consumption as well as prolong network lifetime. However, the hotspot problem cannot be avoided since we try to minimize the total energy consumption during certain route rather than to let sensor nodes consume their energy at similar rate.

Even though the above-mentioned energy efficient routing protocols or algorithms can improve energy efficiency and prolong network lifetime to some degree, they cannot effectively overcome the hotspot problem which is tightly related with energy and traffic models. In this paper, we propose a Distance-based Energy Aware Routing (DEAR) algorithm which can effectively alleviate the hotspot problem based on the theoretical deduction and analysis of relevant models.

## 3. System Model and Problem Statement

### 3.1. System Model

#### 3.1.1. Network Model

The traditional WSNs can be regarded as an undirected graph *G = <V, E>* where *V* represents the set of vertices and *E* represents the set of edges (or links) [9]. Sink node (or BS) can be placed either inside or outside the area to be monitored. We assume that there are *N* nodes randomly scattered in a two dimensional square field [X, Y]. There exists a link *E*(*i, j*) between node *I* and node *j* if the Euclidean distance *d*(*i, j*) is not larger than the radio transmission radius *R*, namely *d*(*i, j*) ≤ *R*. Here, undirected graph means bi-directional communication link. In other words, if node *j* can receive packet from its neighboring node *i*, it is believed that node *i* can receive packet from node *j* in a reverse way. The objective in this paper is to find a set of optimal or sub-optimal individual distances during routing process so that the energy is consumed at similar rate for all involved sensors.

#### 3.1.2. Traffic Model

There are four types of traffic patterns in WSNs, which are time-based, event-based, query-based and hybrid traffic pattern [1]. Usually, routing is trigged when a source node has traffic to send. It is worth noting that traffic pattern has a very important impact on routing performance like energy consumption, latency, *etc.*

Time-based traffic pattern is used when each sensor nodes take turns to report their collected data in a time series manner. It is mainly used in applications like temperature and seismic monitoring where long term observation results like mean value or trend are needed for future prediction. Event-based traffic pattern is used for applications like target tracking or intrusion detection *etc.* When a target is entering into the nearby region of a sensor node, the target will be detected and tracked with an increased (or burst) traffic sent by involved sensor nodes to remote sink node. Query-based pattern means remote commander can send query to obtain observations in certain area. Hybrid traffic pattern is also commonly used. For example, during the time-based traffic monitoring period, the remote sink node may send a query to demand for certain information simultaneously. In this paper, we will study the characteristic of energy consumption under different traffic patterns so as to achieve energy balancing and prolong network lifetime.

#### 3.1.3. Energy Model

**Figure 2.**
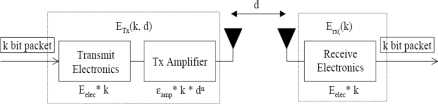
Radio energy dissipation model.

The energy consumption model we use here is called first order radio model [5–8,14], as is shown in Figure 2.

Each sensor node will consume the following *E_Tx_* amount of energy to transmit a *l*-bits message over distance *d*:

(1) $$E_{Tx}(l,d)=\{\begin{array}{ll} l\cdot E_{\mathrm{elec}}+l\cdot {ɛ}_{fs}\cdot d^{2}, & \mathrm{if}\;\;d<d_{0}\\ l\cdot E_{elec}+l\cdot {ɛ}_{mp}\cdot d^{4}, & \mathrm{if}\;\;d\geq d_{0} \end{array}$$

*E_Rx_* amount of energy to receive this message:

(2) $$E_{Rx}(l)=l\cdot E_{\mathrm{elec}}$$

and *E_Fx_* amount of energy to forward this message:

(3) $$E_{Fx}(l,d)=E_{Tx}(l,d)+E_{Rx}(l)=\{\begin{array}{ll} 2l\cdot E_{\mathrm{elec}}+l\cdot {ɛ}_{\mathrm{fs}}\cdot d^{2}, & \mathrm{if}\;d<d_{0}\\ 2l\cdot E_{\mathrm{ele}c}+l\cdot {ɛ}_{mp}\cdot d^{4}, & \mathrm{if}\;d\geq d_{0}. \end{array}$$

The definition of the radio parameters is listed in Table 1.

### 3.2. Problem Statement

Many traditional routing protocols can improve the performance of energy efficiency and network lifetime by introducing intelligent clustering methods or considering residual energy, *etc.* However, most of these energy efficient routing protocols do not consider the energy consumption under various traffic models, which is an important factor that influences the hotspot problem.

**Figure 3.**
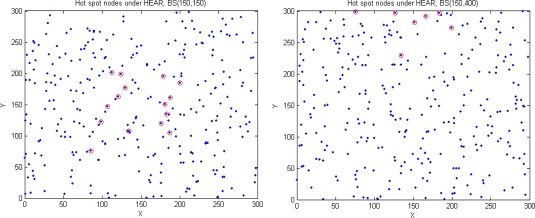
Hop spot nodes under HEAR algorithm **(a)** when BS is inside; **(b)** when BS is outside.

Figure 3 shows the distribution of hotspot nodes with circles under our previous work in [5], where BS is placed inside at (150, 150) in Figure 3(a) and outside at (150, 400) in Figure 3(b). The hotspot nodes are close to the BS since multi-hop routing is favorable in most cases. Even though our HEAR algorithm in [5] can largely reduce energy consumption and prolong network lifetime to certain degree, it cannot avoid the hotspot problem due to its intrinsic nature of minimizing the total energy consumption during certain route. In other words, if we aim to prolong and maximize the network lifetime, the final objective function should be let each sensor consume their energy at similar rate (*E_1_* *≈ E_2_≈* ⋯ *≈ E_n_*) rather than to $\mathrm{Min}(\sum_{i=1}^{n}E_{i})$ for the *n*-hop route.

## 4. Distance-Based Energy Aware Routing (DEAR) Algorithm

### 4.1. Theoretical Analysis of Hotspot Problem

#### 4.1.1. Event-based Traffic Model

**Figure 4.**
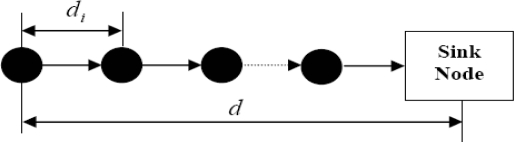
One dimensional linear network.

We first study the one dimensional linear network for simplicity, which can be used in linear applications like highway traffic monitoring, congestion control *etc.* In Figure 4, there are *n* sensor nodes placed along a line from source node to sink node with individual distance {*d_1_*, *d_2_*, ⋯, *d_n_*}. The source to sink node distance is *d* and $\sum_{i=1}^{n}d_{i}=d$.

Once an event is detected by the source node, it will send an *l*-bits message through either direct transmission or multi-hop transmission to the remote sink node. Since multi-hop transmission is more energy efficient when *d* is large, here we study the *n*-hop transmission from source to sink node. Our first objective is to find the optimal multi-hop number and each individual distance *d_i_* so that each node consumes the least energy at similar rate. Based on Equation (1) to (3), the total energy consumption to transmit one bit data (*l* = 1) over *n*-hop route will be:

(4) $$E(n)=(E_{\mathrm{ele}c}+{ɛ}_{\mathrm{amp}}\cdot d_{1}^{\alpha })+\sum_{i=2}^{n}(2E_{\mathrm{ele}c}+{ɛ}_{\mathrm{amp}}\cdot d_{i}^{\alpha })=(2n-1)E_{\mathrm{elec}}+\sum_{i=1}^{n}{ɛ}_{amp}\cdot d_{i}^{\alpha }$$

where, *ε_amp_* = *ε_fs_* when *α* = 2 and *ε_amp_* = *ε_mp_* when *α* = 4. For fixed $\sum_{j=1}^{n}d_{i}=d$, $\sum_{i=1}^{n}d_{i}^{\alpha }$ in Equation (4) has a minimal value when *d_1_* = *d_2_* = ⋯= *d_n_* = *d/n*. Therefore, *E*(*n*) is finally equal to:

(5) $$E(n)=(2n-1)\cdot E_{\mathrm{elec}}+\varepsilon_{\mathrm{amp}}\cdot n^{1-\alpha }\cdot d^{\alpha }$$

Equation (5) has the minimum when *E^′^*(*n*) = 0 or

$$2E_{\mathrm{elec}}+\varepsilon_{\mathrm{amp}}\cdot (1-\alpha )\cdot {\left(d/n\right)}^{\alpha }=0$$

Thus, we can get the final optimal hop number as:

(6) $$n_{\mathrm{opt}}=d\cdot {\left(\varepsilon_{\mathrm{amp}}\cdot (\alpha -1)/2E_{\mathrm{elec}}\right)}^{1/\alpha }.$$

and the corresponding distance [5,22]:

(7) $$d_{i}=d/n_{\mathrm{opt}}=\sqrt[{\alpha }]{\frac{2E_{\mathrm{elec}}}{\varepsilon_{\mathrm{amp}}(\alpha -1)}}$$

Since the distance *d_i_* as well as the traffic length is the same for all sensor nodes, the only difference of energy consumption is *E_Rx_(l)* = *l · E_elec_*, as can be seen from Equation (1) to (3). Therefore, we can get *E_1_* *≈ E_2_* *=E_3_* ⋯ *= E_n_*.

The query-based traffic model is similar to the event-based model in terms of energy consumption since the length of data message as well as the individual distance is the same for all sensors. In both cases, all involved sensors will consume their energy at similar rate, which can effectively alleviate the hotspot problem and prolong the network lifetime.

#### 4.1.2. Time-Based Traffic Model

Under the time-based traffic model, each node will take turns to transmit its data to a sink node. Therefore, the key difference between time-based and event/query-based traffic model is the packet length. For example, node 1 which is the furthest from sink node only needs to transmit its data once while node *n* which is closest to sink node has to transmit its own data once and help forward the data (*n* − 1) times along the line.

It is worth noting that if we use the same methodology to let *d_1_* = *d_2_* = ⋯= *d_n_* = *d/n*, node *n* will become a hotspot node and die quickly since it has more traffic burden to forward. On the other hand, node 1 will have much residual energy when node *n* dies, which is not desirable. Thus, the objective we have here is to let *E_1_* *= E_2_* *=E_3_* ⋯ *= E_n_* in order to maximize network lifetime.

Since node *i* will transmit its own *l*-bits data for once and forward the traffic for (*i* − 1) times after all nodes take turn to transmit their data to sink node. The energy consumption for node *i* is:

(8) $$E_{i}=l\cdot (E_{\mathrm{ele}c}+\varepsilon_{\mathrm{amp}}\cdot d_{i}^{\alpha })+l\cdot (i-1)(2E_{\mathrm{elec}}+\varepsilon_{\mathrm{amp}}\cdot d_{i}^{\alpha })=l\cdot (2i-1)E_{elec}+l\cdot i\cdot \varepsilon_{\mathrm{amp}}\cdot d_{i}^{\alpha }$$

Let *E_i_* = *E_i_*_+1_, we can finally get:

(9) $$d_{i+1}=\sqrt[{\alpha }]{\frac{-2E_{\mathrm{elec}}+i\varepsilon_{\mathrm{amp}}d_{i}^{\alpha }}{\varepsilon_{\mathrm{amp}}(i+1)}}=\sqrt[{\alpha }]{\frac{-2iE_{\mathrm{elec}}+\varepsilon_{amp}d_{1}^{\alpha }}{\varepsilon_{amp}(i+1)}}$$

Since *d_n_* > 0, namely $\frac{-2(n-1)E_{\mathrm{elec}}+\varepsilon_{\mathrm{amp}}d_{1}^{\alpha }}{\varepsilon_{\mathrm{amp}}n}>0$, it must satisfy:

(10) $$n<\frac{\varepsilon_{\mathrm{amp}}d_{1}^{\alpha }}{2E_{\mathrm{elec}}}+1\;\;\;\text{or}\;\;\;d_{1}>\sqrt[{\alpha }]{\frac{2(n-1)E_{\mathrm{elec}}}{\varepsilon_{\mathrm{amp}}}}$$

Based on (10), we can get the corresponding lower bound distance *d*_1_ when hop number *n* = [2:9], as is shown in Table 2. Here, *E_elec_* = 50 *nJ/bit*, *α* = 4 and *ε_amp_* = 0.001 *pJ/bit/m*^4^. Given the multi-hop number *n*, Table 2 provides the lower bound distance value *d*_1_ as well as the minimal source to sink node distance *d* = Σ*d_i_*. For example, when *d* = 300, we can only use 2-hop or 3-hop route to achieve energy balancing with practical distance *d*_1_(2) > 100 or *d*_1_(3) > 118.9. If the hop number *n* ≥ 4, the minimal *d* = Σ*d_i_* ≥ 307.6 which is contrary to *d* = 300.

On the other hand, given the source to sink node distance *d*, there might be several multi-hop routes with different hop number *n*. There exists the highest hop number above with the minimal energy consumption for each sensor node and this is the optimal multi-hop number we need.

From Table 3, we can see different set of *d*_1_(*n*) given the source to sink node distance *d*. For example, when *d* = 800, we can either choose 8-hop route with *d*_1_(8) = 164.8 or choose 7-hop route with *d*_1_(7) = 170.5. The corresponding individual distance *d*_i_ can be deduced from Equation (9). It is worth noting that we cannot choose 9-hop route since the minimal Σ *d_i_* (9) = 840.9 > *d* = 800. Thus, we can choose *n*-hop route with *n* ∈ [2,8] when *d* = 800. Finally, we choose the highest 8-hop route with *d*_1_(8) = 164.8 since each of the 8 nodes consumes the least energy of $E_{i}=E_{1}=l(E_{\mathrm{elec}}+\varepsilon_{\mathrm{amp}}d_{1}^{a}(8))$.

From the analysis under both event/query based and time-based traffic model, we can conclude that:

1. Given the source to sink node distance *d*, the optimal multi-hop number *n* as well as each individual distance *d_i_*, *i* ∈ [1,*n*] can be determined so that all the sensor nodes consume their energy at similar rate;
2. The event or query based model will finally become time-based traffic model when the observing time is long enough. In that case, each sensor node will be almost uniformly chosen for once among all sensor nodes from time point of view, which is similar to the time-based traffic model.
3. Therefore, the time-based traffic model is more popular and practical and we just focus on the analysis of time-based traffic model in the following sections.

### 4.2. DEAR Algorithm

In the DEAR algorithm, each sensor node has two tables. One is the routing table which contains information like source node, previous and next hop node, *etc*. The other table is the neighbor table which contains neighbors’ information like distance between them, distance to the sink node, residual energy, node degree, *etc*. Thus, each node can make intelligent decisions about the next hop based on the DEAR algorithm and the algorithm is easy to implement for practical engineering applications.

The key strength of DEAR algorithm is that given the source to sink node distance d and hardware parameters in Table 1, we can provide energy efficient route with the optimal multi-hop number and corresponding individual distance under the practical sensor network. The energy will be consumed at similar rate for all sensor nodes so that energy efficiency and energy balancing can be achieved and network lifetime can be prolonged.

#### 4.2.1. Basic Assumptions

We make the following basic assumptions similar to [17]:

♦ All sensor nodes are static after deployment.
♦ The communication links are symmetric.
♦ Each sensor node can control its power level to the neighbors.
♦ Each sensor node can know the distance to its neighbors and to the sink node.
♦ We assume ideal MAC layer conditions.

Here, we make no assumption of the uniform distribution of sensor nodes or the knowledge of global network topology. Based on the received signal strength index (RSSI) or other positioning and localization techniques, each node can get the distance to its neighbors and to the sink node and then adjust its transmission power level.

#### 4.2.2. Flow Chart of DEAR

**Figure 5.**
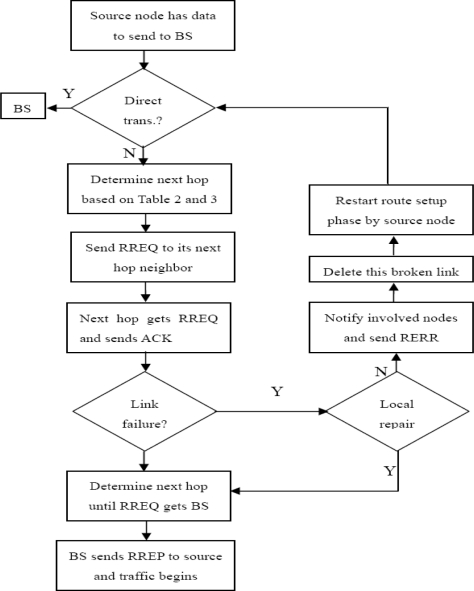
DEAR flow chart.

From Figure 5 we can see that the flow chart of DEAR algorithm consists of two phases. On the left side is the route setup phase and on the right side is the route maintenance phase.

If the ACK message is not received within a certain time, a link failure is detected. Then a local repair process will be initiated first. If the node can find an alternative next hop node, it will determine its next hop in a similar way like above. Or else, it will notify all the involved nodes about the failure of this link by sending a route error (RERR) message. This broken link will be deleted by all involved nodes from their routing table and neighboring table and they will avoid using this link later on. Finally, the source node will restart the route setup phase.

#### 4.2.3. Route Setup Phase

The whole route setup phase is summarized in Algorithm 1. Once source node has data to send, it will try to set up a route from source to sink node as follows.

**Algorithm 1.**
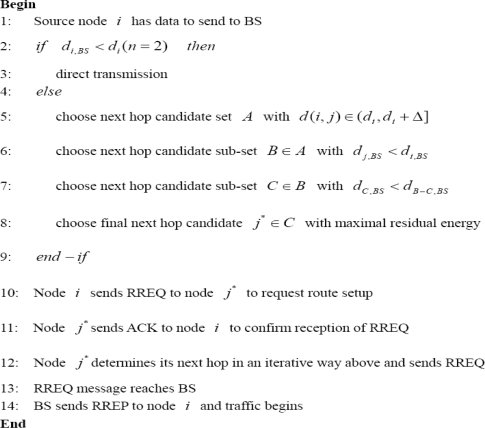
Route setup phase.

First, it determines the transmission manner by comparing the source to sink node distance *d* and *d_i_*(*n* = 2) = 100 in Table 2, as is explained above. It is worth mentioning that we might choose direct transmission when d ≥ 100 + Δ under real network environment. For example, when *d* = 120, it is very difficult to find a 2-hop route with *d_1_* = 100 and *d_2_* *=* 20 so that *E_1_* = *E_2_* under random network topology, especially when BS is placed outside. The value of Δ is dependant on network density and we set Δ ∈ [20,50] in this paper under different network topologies.

Once multi-hop transmission manner is chosen, source node will determine its next hop as is illustrated in Table 2 and 3. It is worth emphasizing that the selection criterion of next hop node is critical during routing process. Here, we choose the final next hop based on the following rules:

♦ The distance between node *i* and its next hop node *j* should be *d* (*i,j*) ∈ (*d_i_*, *d_i_* + Δ].
♦ The distance between node *j* to BS should be less than node *i* to BS, namely: *d_j,BS_* < *d_i,BS_*.
♦ The final next hop node *j* should have relatively much residual energy.

Therefore, the final next hop node *j* is chosen after the following 3 steps. First, we choose a set of candidates with *d* (*i,j*) ∈ (*d_i_*, *d_i_* + Δ] based on the analysis in Equation (9) and Table 2 and 3. Then, some of the candidates whose distance to BS is less than the current node *i* to BS are chosen based on greedy routing mechanism, namely *d_j,BS_* < *d_i,BS_*. Among them, we will choose half of them whose distance to BS is smaller than the other half. Finally, the one with the largest residual energy is chosen as the next hop. It is worth emphasizing that our first parameter is the distance and our secondary is the residual energy in order to achieve energy balancing and energy efficiency during routing process.

When the next hop node is chosen, the source node will send a RREQ message to that specific neighbor directly with its own location information encapsulated inside RREQ. Once the neighbor receives the RREQ message, it will send an ACK message back to its previous node. Then, it will add its own location information again into the RREQ message and send it to its next hop neighbor in an iterative manner like above. Finally, the RREQ message will reach the sink node carrying the complete route information and a RREP message will be sent back to the source node based on the assumption of symmetric link.

The traffic can get started once the source node gets the RREP message with complete route information from sink node. After the traffic session is closed, each node on the route will update its routing table as well as neighboring table.

#### 4.2.4. Route Maintenance Phase

As can be seen from Figure 5, link failure will be detected and route maintenance phase will be initiated if an ACK message is not received for certain TTL (time-to-live) time. Link failure is usually caused by reasons like node dies, interference, *etc.*

If the source node detects a link failure, it will restart the route setup phase by choosing another next hop in a similar way above. If an intermediate node detects a link failure, it will first attempt a local link repair process. In other words, the intermediate node will first try to choose another proper neighbor in order to fix the route to BS. In this way, the end to end latency can get reduced and the energy consumption as well as overhead can be reduced. This local repair process will last for certain time until an ACK message is received or when TTL is expired.

If the local link repair process fails, a RERR message will be sent from intermediate node to source node in a reverse way based on the information stored in RREQ. Finally, this route will be deleted from source node and intermediate nodes and a new route setup phase will be initiated.

## 5. Performance Evaluation

### 5.1. Simulation Environment

For performance evaluation, we use MATLAB software which is similar to C language where the code is executed line by line. As is shown in Table 4, there are 300 sensor nodes randomly deployed in a 300 × 300 m^2^ area WSN with BS placed in the middle of the area. The maximal transmission radius is 150 meters. Each node takes turn to transmit a 2,000 bits message to BS using either direct transmission or multi-hop transmission based on different routing algorithms. We compare our DEAR algorithm with the following three popular routing algorithms which are direct transmission, greedy and maximal residual energy (MRE) algorithms.

### 5.2. Performance Evaluation

**Figure 6.**
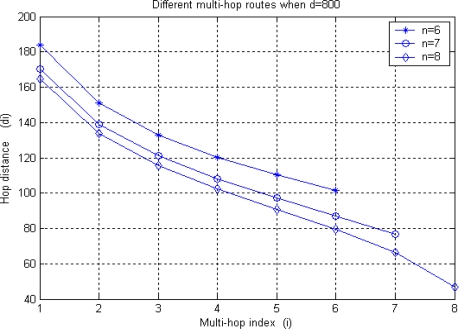
Distribution of hop distance under different multi-hop routes.

Figure 6 shows the distribution of individual hop distance *d_i_* under different multi-hop routes, as is discussed in Equation (9) and Table 2. Given the source to BS distance *d* = 800, there are several multi-hop route options with hop number *n* ∈  [2,8] (see Table 3). Finally, we will choose 8-hop route since each node consumes the least energy. It is worth noting that the practical *d*_1_(8) will be larger than the theoretical value under random network topology. In this way, the energy consumption for all the 8 sensor nodes is the minimal than other *n* hop route where *n* ∈ [2,7]. Therefore, both energy efficiency and energy balancing is achieved.

**Figure 7.**
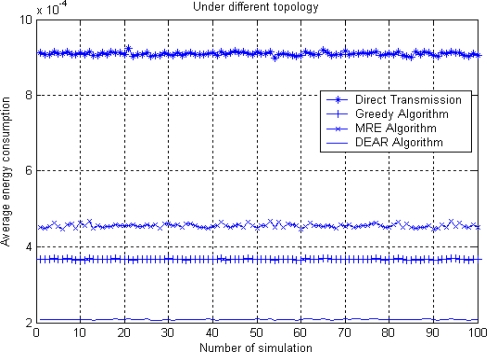
Average energy consumption.

Figure 7 shows the average energy consumption for four routing algorithms under 100 different network topologies. The simulation environment is that there are 300 nodes randomly placed in a 300 × 300 m^2^ area network. We set *R* = 150 and Δ = 40 here.

We can see from Figure 7 that direct transmission algorithm consumes the largest amount of energy since the average source to sink node distance is relatively long and multi-path model is used here. Our DEAR algorithm consumes the least energy due to it distance-based nature. The performance of greedy and MRE algorithm is in the middle under 100 simulations. From here we can see that DEAR can not only balance energy consumption for all sensor nodes but also reduce energy consumption comparing to other algorithms.

**Figure 8.**
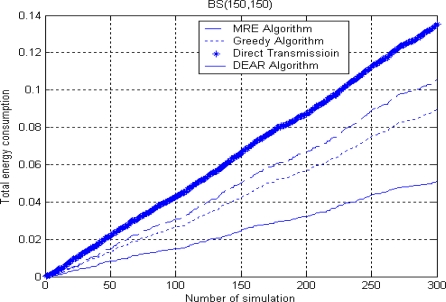
Total energy consumption when BS is inside.

**Figure 9.**
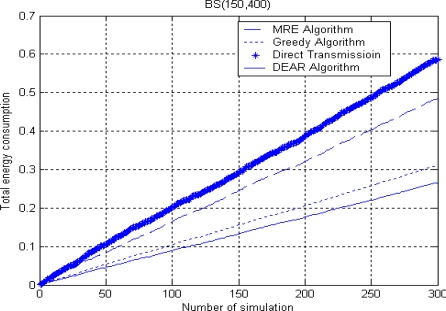
Total energy consumption when BS is outside.

Figures 8 and 9 show the total energy consumption when BS is placed either inside or outside. The simulation environment is similar to Figure 7 where there are 300 nodes randomly deployed in a 300 × 300 m^2^ area. We set *R* = 130 and Δ = 20 here.

From both figures, we can see a similar trend of energy consumption for the four routing algorithms where the direct transmission algorithm consumes the largest energy while our DEAR algorithm consumes the least energy. The total energy means the summation of energy consumption for each algorithm up to the current simulation round. Besides, we can see that the total energy consumption increases sharply when BS is placed outside at (150, 400) since the average source to BS distance is much larger than before.

**Figure 10.**
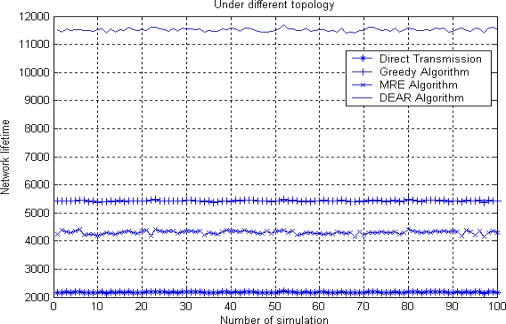
Network lifetime.

Figure 10 shows network lifetime under the same network environment as in Figure 7. Here, network lifetime is defined as the time when the first node dies out of energy since this might cause network partition or isolated area quickly afterwards.

As can be seen from Figure 10, direct transmission usually has the shortest lifetime in a large scale network scenario or when the average source to sink node distance is large. For the MRE algorithm, a node chooses its next hop based on the remaining energy which is irrelevant to the distance distribution. Therefore, the final multi-hop route might has too many short hop number which consumes more average energy, as is shown in previous Figure 7. For a greedy routing algorithm, each node will prefer to choose its next hop with distance close to *R* in order to make greediest progress toward the sink node. Thus, more energy consumption is caused with relatively short network lifetime. Again we can see the DEAR algorithm has the longest network lifetime due to its energy efficiency and balancing nature.

### 5.3. Discussion

Given the source to sink node distance and the hardware parameter values in Table 1, we can determine the transmission manner, the optimal multi-hop number as well as the corresponding individual distances. The key difference between DEAR and other energy efficient routing algorithms is that we try to let each node consume the energy at similar rate rather than to minimize the total energy consumption during each routing process.

The selection criterion of the next hop node is the key problem during routing process. In this paper, we treat the distance metric as the primary parameter and we also consider node residual energy as the secondary parameter. Thus, the energy consumption can be balanced well based on the distance and residual energy distribution. From this paper, we can see that DEAR algorithm is a distributed, localized, energy efficient and balancing algorithm which can be easily used in real applications.

The shortcoming of the DEAR algorithm is the requirement of knowing the source to sink node distance, which can be obtained through GPS devices, certain localization or positioning techniques with additional computing and communication overhead. Also, the DEAR algorithm is not usable in sparse network environments or when there are obstacles between the neighboring nodes. In both cases, the next hop node based on our DEAR algorithm might not be found.

## 6. Conclusions

To efficiently reduce and balance the energy consumption in WSNs, we proposed a Distance-based Energy Aware Routing (DEAR) algorithm based on theoretical analysis and numerical illustration under different energy and traffic models. Given the source to sink node distance, the optimal multi-hop number as well as the corresponding individual distance can be determined so that all sensor nodes can consume energy at a similar rate. During the routing process, we treat distance distribution as the first parameter and the residual energy as the secondary parameter. The final results show that DEAR can ensure better energy efficiency and energy balancing performance comparing with other popular multi-hop routing algorithms.

For future research, we plan to extend our work by studying the influence of hop number and hop distance on other network metrics such as latency, communication overhead, packet delivery ratio, *etc.* Also, we will consider probability based energy efficient routing which integrates in factor of hop distance, residual energy and node degree.

## Figures and Tables

**Table 1.** Definition of radio parameters.

**Parameter**	**Definition**	**Unit**

*E_elec_*	Energy dissipation to run the radio	50 nJ/bit
*ε_fs_*	Free space model of transmitter amplifier	10 pJ/bit/m^2^
*ε_mp_*	Multi-path model of transmitter amplifier	0.0013 pJ/bit/m^4^
*l*	Data length	2,000 bits
*d_0_*	Distance threshold	$\sqrt{\varepsilon_{\mathrm{fs}}/\varepsilon_{\mathrm{mn}}}$m

**Table 2.** Corresponding *d_i_* when hop number *n* = [2:9].

**n**	**d_1_**	**d_2_**	**d_3_**	**d_4_**	**d_5_**	**d_6_**	**d_7_**	**d_8_**	**d_9_**	Σ*d_i_*
2	100.0	0								100.0
3	118.9	84.1	0							203.0
4	131.6	100.0	76.0	0						307.6
5	141.4	110.7	90.4	70.7	0					413.2
6	149.5	119.0	100	84.1	66.9	0				519.5
7	156.5	125.7	107.5	93.1	80.0	63.9	0			626.7
8	162.7	131.6	113.6	100.0	88.0	76.0	61.5	0		733.4
9	168.2	136.8	118.9	105.7	94.6	84.1	73.1	59.5	0	840.9

**Table 3.** *d*_1_(*n*) under different *d*.

d	800	900	1000
*d*_1_(*n*)	d_1_(8) = 164.8	d_1_(9) = 169.7	d_1_(10) = 174.3
	d_1_(7) = 170.5	d_1_(8) = 174.2	d_1_(9) = 177.8
	d_1_(6) = 183.7	d_1_(7) = 184.7	d_1_(8) = 186.3

**Table 4.** Simulation environment.

**Parameter**	**Value**
Network size	300 × 300 m^2^
Node number	300
Radius	150 m
Data length	2,000 bits
Initial energy	2 Joule
*E_elec_*	50 nJ/bit
*ε_amp_*	0.001 pJ/bit/m^4^
_Δ_	[20,50] m
BS	inside or outside
